# Screaming Body and Silent Healthcare Providers: A Case Study with a Childhood Sexual Abuse (CSA) Survivor

**DOI:** 10.3390/ijerph15010094

**Published:** 2018-01-08

**Authors:** Sigrun Sigurdardottir, Sigridur Halldorsdottir

**Affiliations:** School of Health Sciences, University of Akureyri, 600 Akureyri, Iceland; sigridur@unak.is

**Keywords:** child sexual abuse (CSA), female CSA survivors, psychological trauma, disclosure, women’s health, healthcare providers, chronic illness, case study, phenomenology, interviews

## Abstract

Stressful early life experiences cause immune dysregulation across the lifespan. Despite the fact that studies have identified childhood sexual abuse (CSA) survivors as a particularly vulnerable group, only a few attempts have been made to study their lived-experience of the physical health consequences of CSA. The aim of this study was to explore a female CSA survivor’s lived-experience of the physical health consequences of CSA and how she experienced the reactions of healthcare providers. Seven interviews were conducted with this 40-year-old woman, Anne, using a phenomenological research approach. Anne was still a young child (two to three years old) when her father started to rape her. Since her childhood, she has experienced complex and widespread physical health consequences such as repeated vaginal and abdominal infections, widespread and chronic pain, sleeping problems, digestive problems, chronic back problems, fibromyalgia, musculoskeletal problems, repeated urinary tract infections, cervical dysplasia, inflammation of the Fallopian tubes, menorrhagia, endometrial hyperplasia, chlamydia, ovarian cysts, ectopic pregnancies, uterus problems, severe adhesions, and ovarian cancer. Anne disclosed her CSA experience to several healthcare providers but they were silent and failed to provide trauma-informed care. Anne’s situation, albeit unique, might reflect similar problems in other female CSA survivors.

## 1. Introduction

There is no real distinction between mind and body because of the communications between the brain, the nervous system, endocrine system, and immune system [1]. Psychological trauma, such as after childhood sexual abuse (CSA), has long-term physical consequences because stressful early life experiences cause immune dysregulation across the lifespan [2]. This means that individuals with CSA experience are at a greater risk for serious illnesses than those without such experience. Table 1 [3,4,5,6,7,8,9,10,11,12,13,14,15,16,17,18,19] shows the known physical consequences of CSA. 

Table 2 [20,21,22,23,24,25,26,27,28,29] is an overview of the prevalence of CSA in some countries. It shows that approximately one in every four women (10.2–40.2%) has experienced CSA. This suggests that healthcare providers, especially in high volume practices, encounter multiple women who are CSA survivors every day.

As Table 1 shows, many physical consequences of CSA are known. These include widespread and chronic pain, sleeping problems, adult onset arthritis, fibromyalgia, long-term fatigue, diabetes, and circulatory-, digestive-, respiratory-, and musculoskeletal problems, as well as reproductive and neurological ones. Moreover, survivors often develop medically unexplained symptoms [5] which go hand in hand with significantly higher healthcare use such as a greater number of visits to the emergency department, hospital outpatient department, pharmacy, primary care, and specialty care than those without a CSA experience [3,30,31].

The emotional and psychological part of the body suffers in traumas and CSA survivors often deal with complex mental problems that greatly affect their well-being and health [32,33,34]. Adult CSA survivors often deal with symptoms such as anxiety and depression, dissociation, numbing, freezing, repressed memory, sleep disturbance, and self-mutilation [35,36,37,38], as well as sexual dysfunctions [39]. CSA can affect people’s coping strategies and lead to PTSD (Post-traumatic stress disorder) [40]. It accounts for more variance in PTSD than adult sexual assault [41] and CSA survivors are at a great risk of developing complex PTSD [42]. The experience of dissociative symptoms after a trauma event increases the risk of developing PTSD [43]. 

Despite the fact that studies have identified CSA survivors as a particularly vulnerable group, only a few attempts have been made to study their lived-experience of the physical health consequences of CSA. No study was found where a CSA survivor’s life story is examined for a holistic view of the physical health consequences. Since there are indications of gender differences in the CSA experience, men reacting with extroversion and women with introversion, which seems to lead to more health problems for female CSA survivors [44], we decided to explore the lived experience of physical health consequences of a female CSA survivor. The research question was twofold: what is the lived experience of a female CSA survivor of the physical health consequences of CSA, and what is her experience of the reactions of healthcare providers? The reason for the latter part of the research question is that we wanted to know if healthcare providers had recognized and validated her lived-experience as a CSA survivor and the connection these experiences might have on her physical health. 

## 2. Materials and Methods

The research method was a phenomenological case study using the Vancouver School of doing phenomenology, a unique blend of phenomenology, hermeneutics, and constructivism [45]. It is one of the few Ricoeurian phenomenological schools and is popular among researchers in the Nordic region [46]. A case study allows the examination of complex cases to deepen our understanding of unique phenomena [47], a research method involving an up-close, in-depth, and detailed examination of a subject of study [48]. It can be an analysis of a person and a person’s life that is studied holistically [49]. Similar to most studies founded on the phenomenological tradition, this study is based on the premise that reality is individually constructed as a result of lived experience. Within phenomenology, emphasis is placed upon seeing all individuals in their context, as well as the understanding that each person perceives the world in a unique way, and that individual perception is molded by former experience and the interpretation of that experience [50]. The method is thus person-centered; the inquiry aims to produce understandings from the point of view of the individual. The researcher must make sense of the data in a meaningful way, along with the research participant, who is seen as a dialogue partner and co-researcher [45]. The research process in the Vancouver-School is shown in Table 3.

Step 1 in a Vancouver-School phenomenological case study is selecting the dialogue partner. Eligibility for participation in the current study was having experienced CSA and a willingness to talk about it. The volunteer research participant came from a group of 12 female CSA survivors who took part in The Wellness Program [51], a ten-week organized program for female CSA survivors. For the sake of anonymity, she is called Anne. She is a 40-year-old female CSA survivor.

Step 2 is silence. Because the study is a process, this reflective silence was re-entered repeatedly throughout the study [45]. In the silence, the primary researcher reflected on preconceptions about the phenomenon to identify their own ideas and thoughts on the subject. Throughout the study, the primary researcher kept a reflective journal of her thoughts about the study.

Step 3 of the Vancouver School involves in-depth interviews. Data collection was conducted through individual interviews. The first author, as the primary researcher, conducted all the interviews. Each interview took 30 to 60 min. Data collection stopped when no new information was obtained as judged by the primary researcher. During the interviews, the primary researcher used an interview guide but strived to ask open questions and encourage Anne to express herself freely, also seeking confirmation of emerging themes and probing further into individual topics arising during the interviews. Interview questions were for example: Can you tell me about your experience of violence? How has your health been affected by the violence? What is your experience of communicating with healthcare providers regarding CSA? Did you experience support from them as a CSA survivor? The primary researcher interviewed Anne seven times. The interviews were aimed at exploring Anne’s life story concerning the physical health consequences of CSA and how healthcare providers reacted to her as a CSA survivor. 

Steps 4–6. The interviews were recorded, transcribed, and analyzed for main themes and subthemes through thematic analysis. Codes were extracted from the transcripts (deconstruction). Those were then arranged into themes (reconstruction). The findings were constructed into an analytic framework, in accordance with Steps 4 through 6. The primary researcher repeated this procedure after each interview until a holistic understanding of Anne’s life and physical health was constructed. 

Step 7. To ensure that her words were understood correctly, Anne verified her story after each of the interviews. This verification process is one of the main strengths of the Vancouver-School because the participant is able to verify or correct the researcher’s interpretation about the participant’s lived-experience.

Step 8. After the primary researcher’s initial work, both researchers were involved in the data analysis, synthesis, and interpretation of the data. The primary researcher discussed the preliminary findings with the second author. All possible variations were explored independently and together, and after much deliberation, the essential structure of the lived-experience of the physical consequences of CSA and the reaction of healthcare providers to her as a CSA survivor were constructed. 

Step 9. The primary researcher then ensured that this interpretation of Anne’s story was based on the actual data by rereading all the transcripts and comparing them with the findings. 

Step 10 is about constructing the over-arching theme of the study. Just as the research question was twofold, so is the overarching theme as constructed by both authors, “Screaming body and silent healthcare providers”, which was deemed the essence of the physical health consequences of CSA, as well as the reactions of healthcare providers—from the perspective of Anne as a CSA survivor. 

Step 11. Anne then verified the results and the conclusions in a face-to-face meeting. 

Step 12. In writing up the findings, Anne was quoted directly to increase the trustworthiness of the findings and conclusions. 

**Figure 1:**
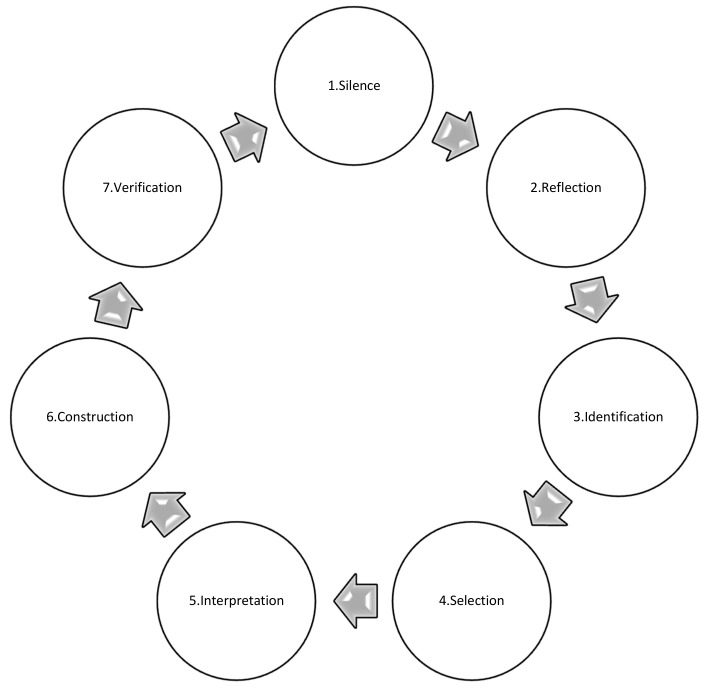
The process of doing phenomenology in the Vancouver School [45]. In every step of the 12 steps of the Vancouver School, this cycle is repeated.

At every step, the researchers went through the research process of the Vancouver school: silence, reflection, identification, selection, interpretation, construction, and verification (see Figure 1).

### 2.1. Ethical Aspects of the Study

Before the first interview commenced, Anne was given an introductory letter in which all the main ethical aspects of the study were addressed. The first author described the study before the interviews started and Anne signed a document providing her informed consent. The letter of introduction provided her with names of a psychologist and an emergency nurse who had consented to be available for her if difficult emotional reactions emerged during or after the interviews. The time between the interviews was about two to six weeks, which gave Anne an opportunity to reflect on the experience of the former interviews. Because the study involved a vulnerable woman, every effort was made to protect her ethically. In order to protect her personal identity, tape recordings were deleted as soon as interviews had been transcribed; and all information that could identify her was removed from the transcripts. Ethical permission was obtained from the National Bioethics Committee (VSNb2011080004/03.7) and the study was reported to the Data Protection Authority (S7663/2016/TS/). 

### 2.2. Validity and Reliability

Each interview was analyzed thoroughly, emphasizing critical assessment of the quality of data collection, data analysis, and presentation of findings. The research process of the Vancouver School has some in-built strategies designed to enhance validity, particularly “member checking” in steps 7 and 11 (see Table 3). The “researcher triangulation” in this study proved fruitful, especially in steps 8, 10, and 12. Triangulation is one of the strategies designed to enhance validity and reliability in qualitative research. “Peer debriefings” and “thick description” were also used as strategies to enhance validity [45]. The findings are a construction of the researchers, based on the data. A “reflective diary” was used at all stages of the research process.

## 3. Results

Similar to the research question, the overarching theme of the study was twofold, “Screaming body and silent healthcare providers”. On one hand, it denotes the multitude of physical complications, need for medical treatment and chronic pain, and discomfort and ailments Anne has been dealing with all her life (screaming body). On the other hand, it answers the question of how she experienced healthcare providers’ reactions (silent healthcare providers). It thus answers the research question about the lived-experience of the physical health consequences of CSA, as well as the reactions of healthcare providers, who were silent and did not provide her in any way with trauma-informed care. In presenting the findings, we first give an overview of Anne’s story to provide the context of her lived-experience. Then we present her experience of the burden of CSA on her body and the reactions of healthcare providers to her as a CSA survivor. 

### 3.1. Anne’s Story

When Anne was eight months old, her mother remembers something suspicious about her father regarding Anne. Anne was still a young child (two to three years old) when her father began to rape her and he did that until she was nine years old when he had the chance. She later brought charges against her father. He pleaded guilty but the charges were dropped because they were considered too old for prosecution. Anne was also raped by her uncle, her stepfather, her friend’s father, and by more than one relative. An overview of Anne’s life is found in Table 4, denoting her main psychological traumas and main physical health problems. 

Anne has had symptoms of anxiety since childhood, suffered from depression and social anxiety since she was a teenager, and has suffered from severe postpartum depression. She has suffered from flashbacks, nightmares, numbness and dissociation, a broken self-image, problems with personal boundaries, and repeated re-traumatization. She tried to forget her painful childhood experiences but began having flashbacks when she was 15 years old. She experienced violent nightmares, which intensified when she became sexually active. Anne claims that she was able to endure the abuse by disconnecting herself. She exited her body through dissociation.

“I clearly remember when I was younger, maybe not right when the abuse was happening, but just afterwards—I would lie somewhere and escape to that feeling… I would always exit on the left side, I just left there, it wasn’t a conscious decision. It felt like I was asleep, but I was not sleeping. It didn’t matter what was happening, I just disconnected completely”.

Anne disconnected from herself and her environment, trying to be invisible. She asserts that sexual abuse at such a young age from such a close relative has had a horrendous effect on her and that she has difficulty defining her own and others’ boundaries. From early on in her childhood, her understanding is that her own personal boundaries became blurred, which made her vulnerable to repeated abuse. She was 20 years old when she gave birth to her first child and, shortly after, a relative raped her. She became severely depressed and lost the will to live. Subsequently, she lost the custody of her child. Anne has been in six cohabitations, three marriages, is newly divorced, and has entered a new cohabitation. She has three children with three different men. 

### 3.2. The Burden of CSA on Anne’s Body

Anne has suffered much in her body as long as she can remember. She has suffered from numerous physical health problems such as repeated vaginal and abdominal infections, widespread and chronic pain, sleeping problems, digestive problems, chronic back problems, fibromyalgia, musculoskeletal problems, repeated urinary tract infections, cervical dysplasia, inflammation of the Fallopian tubes, menorrhagia, endometrial hyperplasia, chlamydia, ovarian cysts, ectopic pregnancies, uterus problems, severe adhesions, and ovarian cancer. All of these problems are unknown in her family, except for her 19-year-old daughter who has been diagnosed with fibromyalgia. Anne’s family members are healthy and live a long life. Her grandmother died last year at the age of 90.

### 3.3. Childhood Physical Health Consequences

Anne’s physical symptoms began after her parents divorced and she was sent to her abusive father during weekends. She became very sick during those visits and was sent back home to her mother, where she quickly got better. From early on in her childhood, she was a regular patient in hospitals because of pain, muscle aches, and uterine problems. As a child, she often had stomachaches. During her teens, Anne’s poor health got even worse. She recounts,

“When I was about 12–13 years old, the winter that my mother became pregnant with my sister and my stepfather began making sexual advances to me, I lost nearly all my sight and hearing for a while. My eardrums were perforated and I had a constant ear infection, just from stress”.

### 3.4. Adolescent Physical Health Consequences

During adolescence, she suffered from gastrointestinal problems, an eating disorder, musculoskeletal health problems, genital/urinary problems, and musculoskeletal health problems, as well as severe and chronic uterine pain.

Gastrointestinal problems. As a child, Anne regularly had stomachaches. At age 13, she had an operation due to suspected appendicitis, which turned out to be gastritis: “It was all because of the stress, you see, all these years and you could never talk about it”. She also began to have colon spasms when she was in adolescence. 

Eating disorder. Anne has been overweight since her teens: “I was quite thin as a child but I gained a lot of weight at 16 due to the abuse and my broken self-esteem. I just started eating to numb myself, I didn’t think about what I ate, and I’ve been overweight since then.” She was able to lose 15–16 kg in rehabilitation, but regained the weight after yet another setback. Her weight gain has continued.

Musculoskeletal health problems. Anne has had back problems for as long as she can remember. At sixteen, she had severe myositis (muscle pain) in all her muscles. 

Genital/urinary problems. From an early age, Anne has had repeated urinary tract infections and chronic ovarian cysts since she first became sexually active.

“When I was 16–17, I started getting ovarian cysts, it was chronic… if things got bad, emotionally or stress in my personal life I got ovarian cysts and adhesions. It started as soon as I started having sex at around 16”.

She has also had severe and chronic uterine pain after she became sexually active:

“I was 17 with the father of my first child and all of a sudden I just fell down the day after we had sex, I felt excruciating pain in my uterus. I was sent to hospital by ambulance. There was always pain on the right side of my uterus”.

Sexually transmitted diseases. Anne was first diagnosed with chlamydia at 17, and was diagnosed again so many times after that she was constantly getting antibiotics.

### 3.5. Adult Physical Health Consequences

Anne’s adult physical health consequences have included numerous genital/urinary problems, gastrointestinal problems, musculoskeletal problems, insomnia and fibromyalgia, as well as endocrine problems. She has had numerous surgeries in her adult years.

Genital/urinary problems. At the age of 27, after the birth of her second child, the repeated urinary tract infections returned after sex: “It’s a really intense pain in the stomach that goes up the back and down into the groin area. It’s unbearable”. Once she was sent to hospital with abdominal pain and a serious urinary tract infection and inflamed Fallopian tubes: “I got a horrendous bladder infection. I called the hospital and then I started having heavy menstrual bleeding for ten days, very painful menstruations. I knew that something wasn’t right”. At the age of 34, she had serious problems with her ovaries due to ruptured cysts. She had a hysterectomy when she was 37 years old because of nodules, heavy bleeding (menorrhagia), pain, and endometrial hyperplasia. Anne has had two ectopic pregnancies, when she was 24 and 32. The latter ectopic pregnancy was very difficult for her and she became very depressed. By the age of 32, she had given birth to three children. Anne was about 30 when first diagnosed with cervical dysplasia and went through cervical conization. She had bleeding problems after that. She connects her physical condition partly to the fear, which she kept inside: “I am pretty sure that such intense fear of a person I was so close to has played a part in this”. 

Negative birth experiences. When Anne gave birth to her first child at 20, she experienced no understanding or support from healthcare professionals and for her it was a terrible experience. This happened again when she was 27 and gave birth to her second child. At that time, she had a chlamydia infection, which was passed on to the child, who became very sick. Anne also suffered from repeated abdominal infections as a result.

“I nearly lost everything here on my right side: my ovary and Fallopian tube, it (the infection) was spreading everywhere. I was very sick that whole year. There was always something wrong somewhere (in the body)–(even) infection in my kidneys. The whole system had broken down”.

Gastrointestinal problems. She also suffered from colon spasms. The gastroscopies and X-rays taken of her colon revealed that she had gastric reflux and esophagitis. Anne saw many urologists and family doctors because of her gastrointestinal problems. 

Musculoskeletal problems. Anne has not been able to work since her early thirties because of the pain in her back, lumbar and sacral region, shoulders, and head. “I have had back pain for as long as I can think back, from the lower back to the head”. The pain was so intense that she had difficulty walking and holding onto objects.

Insomnia and fibromyalgia. Anne began suffering from insomnia due to chronic body aches and was diagnosed with fibromyalgia at the age of 33. She states that these symptoms, starting in her teens, included widespread pain, muscle aches, fatigue, and depression, and had become worse in the last few years. 

Other physical health problems. Since Anne turned 35 years old, she has also had symptoms of arrhythmia. She consulted a cardiologist, received a Holter, and had all kinds of tests, but everything seemed to be normal. There was seemingly no explanation for her symptoms. At 39 years of age, an ultra-sound of her neck revealed she had a growth on her parathyroid gland and she was diagnosed with para-thyroid adenoma. 

Surgeries. Anne has had many surgeries. She has had an appendectomy, cervical conization, and three operations to remove adhesions—twice when she was 27 and then again at 28. Her right ovary was operated on, but it was not removed entirely. The pain went away for a while after the operations. At 36, she had surgery to empty an ovarian cyst. She also had laparoscopic surgery because of another ovarian cyst on her right ovary. When the decision was made to remove her uterus (hysterectomy), she specifically asked that her right ovary be taken as well due to the discomfort it had caused her since her early teens. She wanted to get rid of it. The doctor agreed to do so after much deliberation, and Anne had curettage.

“When I regained consciousness, he told me that my uterus was full of nodules (…) I tell him to take them, and when you have opened me up, remove the right ovary. He said ‘no’, I said ‘yes’! ‘I have been in pain in my right ovary since… my girl was born. You open me up and you take it out’, and fortunately he did it… I could feel that something was wrong there”.

After the removal and biopsy of the right ovary and the Fallopian tube, it turned out they were malignant. At that point, the left ovary and Fallopian tube were also removed in another operation shortly after.

“The doctor told me after the operation that it looked good enough, nothing wrong with it. He said that if I hadn’t been so insistent, he wouldn’t have removed it… but seven days later I’m called in to the hospital because they had found first stage cancer in it… The doctor told me, ‘thank you for insisting and pushing me to do it, because within two years you would have been gone, it wouldn’t have been detected during that time’.”.

The cancer was limited to the ovaries, so Anne did not require chemotherapy, but she goes for a check-up every six months.

“But it’s always such a huge pain to go in for these check-ups. Today I need to go and be checked for cancer… It is always just as painful. As time goes by, I’ve learned not to be there while it’s happening, I just ‘go out’, but this is just one of the many factors that may push women not to get checked, which increases the risk of cancer—it brings all kinds of bad feelings to the surface”.

Anne went through a major operation at 40, a para-thyroidectomy. Many tests were done and she was sent to an endocrinologist who diagnosed her with abnormal growth of the parathyroid gland.

### 3.6. Silent Healthcare Providers

Anne has looked to the healthcare system for solutions to her physical health problems but believed that she was not offered treatment solutions appropriate for CSA survivors. As a child, she went with her mother between healthcare providers, crying in pain. As an adult, she visited several healthcare providers and persisted in sharing her thoughts about how her physical problems were related to CSA but their reaction was silence. They failed to provide any consideration, or even validation, of her traumas. Health providers’ solution for her problems was mostly medication: “I’ve been using a lot of pain medication, but I’m now trying not to take them unless strictly necessary”. Anne received many antibiotics for her infections, and a lot of medication for insomnia, pain, and inflammation. She had many demoralizing encounters with healthcare providers offering her psychiatric drugs. Even though she tried to talk about the CSA as a possible source of her many health problems, she was not heard, and there was only silence. Once when Anne met with a gynecologist she told him she was convinced that the sexual violence she suffered in her childhood caused the terrible effects she had to deal with today. She recounted:

“Once I told my gynecologist that I was always in such pain because of my womb, especially after sex, and I told him that it must have been because I was damaged back then when I was so small and sensitive down there, but I never got an answer”.

No one asked her about trauma or CSA and even if she brought it up, she was met with silence.

“When I was diagnosed with fibromyalgia, I… said that it was probably the result of stress in my teens. The doctor asked why I thought it was acquired, and I answered that I had been sexually abused as a child… and there was just silence, and we didn’t discuss it any further”.

### 3.7. The Wellness-Program—A Turning Point for Anne

Anne has constantly been looking for pathways for healing. She has tried writing, singing, painting, praying, and going to self-help meetings and various religious congregations. She has also tried going to rehabilitation programs, e.g., programs for obesity and pain, as well as all kinds of non-traditional therapies. When Anne participated in the Wellness-program [51], a 10-week program specially tailored to female CSA survivors, it was a turning-point for her and changed her life. She noted:

“I had to learn to set boundaries. I needed to do exercises on my own in setting boundaries. I had to look at other people and notice for example how people acted in a crowd. I realized that personal space was something I did not know and had to create. Of course, I still make mistakes. I set boundaries regarding individuals who do not understand them, which I do not even understand myself. Sometimes I push people too far away from me or hurry to close myself, but I have been practicing”.

### 3.8. Anne’s Advice to Healthcare Providers

Anne frequently visited gynecologists due to pain in the uterine region. She regularly had to go to hospital because of the pain. She said that in all her encounters with healthcare providers, she was never asked about sexual violence and she sees that as the main problem: “I think it’s important to ask, to say the bare minimum… you should be able to just put an x somewhere on a form to say that you’ve suffered abuse or violence. That’s always the first step”. Anne stated that although people may not self-identify as CSA survivors at first or even the second time, some eventually would. She believes it is critical that healthcare providers who work with her know her story: “When you go to the doctor for example, a gynecologist, a therapist, a psychologist, they need to know your story. It’s a whole other story to get a doctor who doesn’t know your story… a totally different understanding and interaction”. Anne asserts that the most important aspect of them all is to be able to talk to healthcare providers, and that they in turn believe what you tell them.

“The most wonderful thing was finding people who believed me. It’s all connected, it’s all about telling people your story and expressing yourself, being believed; what you say is heard. Somehow, it makes you feel complete. It’s not about pity (…), just being believed and like I said, to feel relieved. When someone recognizes that it’s not your fault. When you don’t tell your story, you are taking responsibility for what happened. ‘This was my entire fault so I won’t say a word’. When you tell your story, it’s no longer your fault; it’s someone else who should be ashamed”.

## 4. Discussion

In this case study, we explored the physical consequences of CSA by describing the lifetime physical health problems experienced by one woman. The unique findings clearly depict the burden of childhood sexual abuse on the body. The study also demonstrates how healthcare providers failed to recognize and validate her lived-experience as a CSA survivor, as well as the connection these experiences have had on her physical health. The silence she experienced at the hands of healthcare providers is unacceptable. However, it points to a systematic organizational problem in healthcare providers’ education and socialization about the body and mind being separate and subsequently treating only symptoms with medication, not the underlying problem. It also points to an epistemological dualism that implies a primacy of medical knowledge so that if there is not obvious objective evidence known to the healthcare provider justifying the patient’s suffering, the problem is classified as psychogenic. Regrettably, this does not lead to a solution of the problem, and these healthcare users often have to add to the burden of their pain the weight of the delegitimization of their suffering [52].

### 4.1. Reflecting on Anne’s Story

Anne has been suffering from widespread and complex health problems since her childhood, sometimes without any medical explanation. She repressed her memories from the sexual violence, felt frozen and numb, and began having flashbacks and violent nightmares when she got older. When she became sexually active, she disconnected herself from her body and her feelings because of the pain inside. As other studies show, dissociation is a factor that people use when experiencing trauma, a defense mechanism that protects people from suffering [53,54], a long-term coping strategy [55] that can, however, increase the risk of developing PTSD [43]. Anne has symptoms of PTSD even though she has not been diagnosed, similar to other survivors of CSA [40,41,42]. Approximately 70% of female help-seeking victims of sexual assault experience significant levels of traumatization, with 45% reporting symptoms consistent with a probable PTSD diagnosis [56]. When looking at the relationship between the immune- and neuroendocrine systems, we can see how the immune system plays a central role in PTSD, as well as in illnesses found along with PTSD [57]. Anne has had anxiety and depression most of her life and that is very common among CSA survivors [38]. 

### 4.2. Anne’s Suffering Body

Anne has suffered much in her body as long as she can remember. She has had widespread and chronic pain since her childhood, symptoms similar to the physical illness burden presented in other studies [4,6,17,19]. She has been diagnosed with fibromyalgia and has not been able to work since her early thirties because of musculoskeletal problems. She has had symptoms of arrhythmia and was diagnosed with parathyroid adenoma. These are similar findings to other studies regarding cumulative childhood stress and autoimmune diseases in adults [11,12]. Earlier studies indicated that consequences of CSA included symptoms such as eating disorders [12,58] and obesity [59,60], also true in Anne’s case. 

Research results indicate that women with a history of CSA have problems in three domains of the pelvic floor: micturition, defecation, and sexual function [61]. Anne has suffered much in the pelvic area. She has been diagnosed with repeated urinary tract infections, abdominal pain after she became sexually active, and had chronic ovarian cysts, as well as inflammation of the Fallopian tubes. She was diagnosed with cervical dysplasia, had ectopic pregnancies, a hysterectomy, heavy bleeding (menorrhagia), pain, and endometrial hyperplasia. She was diagnosed with chlamydia and suffered from repeated abdominal infections after giving birth. She connects her physical problems to an inner fear, one that she still found hard to talk about. Early life stressors can truly make people more vulnerable to immune dysregulation in adulthood [2] and they are at a greater risk of serious illness [14,62]. CSA increases the risk for re-victimization in adulthood [7,63,64], which is the case in Anne’s life. The CSA has affected Anne’s ability to define her own personal boundaries and made her vulnerable to repeated violence and we need to understand these blurred boundaries further. Anne regularly had stomachaches as a child and had surgery due to gastritis. She has also suffered from colon spasms through the years, has had X-rays, colon and gastroscopies, and gastric reflux and esophagitis. Anne has also had many cardiac symptoms, consulted a cardiologist, and received a Holter (a battery-operated portable device that measures and records the heart’s activity (ECG) continuously for 24 to 48 h or longer). She had ultra-sounds of her heart and other tests indicating that there was seemingly no medical explanation for the symptoms. This lack of medical evidence is consistent with the findings of other studies about medically unexplained physical symptoms in adult survivors of CSA [5,7,9,14]. 

### 4.3. Anne’s Sincere Help Seeking

Anne has constantly searched for ways to live with the physical consequences of CSA. From a young age, she looked to the healthcare system for solutions without receiving adequate help, support, or understanding. Anne feels she not only did not get the support she needed within the healthcare system, but her lived-experience was neither recognized nor validated. Research results by McClure et al. [65] and Putnam [66] show that support is one of the most important treatments for female CSA survivors. Social support also plays a significant role in mediating and moderating some long-term consequences [67]. Anne has consistently asked her healthcare providers to recognize and validate her lived-experience as a CSA survivor, as well as the connection these experiences have had on her chronic health conditions. However, the silence she experienced points to a systemic organizational problem in the education of healthcare professionals and healthcare professional socialization—treating only symptoms and not the underlying problem. Anne has been to health providers multiple times and received much medication for her symptoms. These findings are supported by another study [30], which shows that CSA survivors have significantly higher annual healthcare use and costs than those without a CSA experience. Anne is convinced that the symptoms of fibromyalgia were caused by emotional inner stress after CSA. She told her doctors about the CSA but they were silent, her lived-experience of sexual violence was not discussed any further, and she never got an answer, even though she went regularly to a hospital because of pain after having sex. It is important that healthcare professionals who work with CSA survivors know each survivor’s story to understand the impact of CSA on their body. As Anne said, she has never been asked about CSA or sexual violence and maintains that the first step is to ask, or to be able to just put an x somewhere to say that you have suffered CSA. Even though people may not self-identify as being a CSA survivor at first or even the second time, at least there is the possibility when asked. Anne asserts that what is most important is to be able to talk to people about the CSA, and be listened to, believed, and supported as a CSA survivor. 

### 4.4. Ideas for Further Research

The blurred lines of the boundaries that Anne experienced because of her CSA-experience need to be examined further, especially in terms of how we can shift the boundaries of CSA survivors to safeguard them against future predators. In Anne’s case, there seems to have been a lack of knowledge in how to assess for CSA and support her as a female CSA survivor within the healthcare system. This seeming lack of knowledge needs to be studied further. Moreover, it needs to be studied further how additional sexual assaults affect CSA survivors. Finally, the lifetime sequelae of CSA need to be studied among a larger sample of survivors. 

### 4.5. Limitations and Strengths

The participant in this study is only one woman and we cannot say that she is the most typical of female CSA survivors. Therefore, we cannot generalize from this study to all women who are CSA survivors, but it provides an increased knowledge and deeper understanding of the physical health consequences of CSA and the reactions of healthcare providers to her as a CSA survivor. By using the Vancouver School with its case-study characteristics, we were able to portray the detailed history of Anne’s CSA experiences and the consequences for her body. 

## 5. Conclusions

The results of the study confirm that CSA can have serious and widespread physical health consequences and illustrates the complexity of CSA and its lasting effects. According to Anne, it is important to ask CSA survivors about CSA, provide information about possible consequences, have an understanding, be supportive, and listen with attention. In her encounters with healthcare providers, Anne often felt silenced, marginalized, and re-victimized in a healthcare system that seemed to refuse to validate her lived-experience of CSA and how this is tied to her chronic health problems. This seems to reflect a need for trauma-informed education and training within healthcare educational institutions. 

## Figures and Tables

**Table 1:** Known physical consequences of CSA.

Physical Consequences	Studies
Widespread and chronic pain	Leserman and Drossman, 2007 [3]; Levine, 2010 [4]; Nelson et al., 2012 [5]; Paras et al., 2009 [6]; Wegman and Stetler, 2009 [7].
Sleeping problems	Chapman et al., 2011 [8]; Sigurdardottir and Halldorsdottir 2013 [9]; Wegman and Stetler, 2009 [7].
Adult onset arthritis	Sigurdardottir and Halldorsdottir, 2013 [9]; Von Korff et al., 2009 [10].
Fibromyalgia	Dube et al., 2009 [11]; Sigurdardottir and Halldorsdottir, 2013 [9]; Wilson, 2010 [12].
Long-term fatigue, diabetes	Romans et al., 2002 [13]; Sigurdardottir and Halldorsdottir 2013 [9].
Circulatory problems	Kendall-Tackett, 2009 [14]; Romans et al., 2002 [13]; Sigurdardottir and Halldorsdottir, 2013 [9]; Wegman and Stetler, 2009 [7].
Digestive problems	Dube et al., 2009 [11]; Kendall-Tackett, 2009 [14]; Kuhlman et al., 2012 [15]; Leserman and Drosman, 2007 [3]; Levine, 2010 [4]; Nelson et al., 2012 [5]; Paras et al., 2009 [6]; Wegman and Stetler, 2009 [7]; Wilson, 2010 [12].
Respiratory problems	Anda et al., 2008 [16]; Dube et al., 2009 [11]; Talbot et al., 2009 [17]; Wegman and Stetler 2009 [7]; Wilson, 2010 [12].
Musculoskeletal problems	Sigurdardottir and Halldorsdottir, 2013 [9]; Talbot et al., 2009 [17]; Wegman and Stetler, 2009 [7].
Reproductive problems	Beck et al., 2009 [18]; Paras et al., 2009 [6]; Seith and Teichman, 2008 [19]; Sigurdardottir and Halldorsdottir, 2013 [9].
Neurological problems	Beck et al., 2009 [18]; Kendall-Tackett, 2009 [14]; Paras et al., 2009 [6]; Seth and Teichman, 2008 [19]; Wegman and Stetler, 2009 [7].

**Table 2:** The prevalence of CSA in some countries.

Countries	Women	Authors	CSA Age before	Research Design	Sample
Worldwide 22 countries	19.7%	Pereda et al., 2009 [20]	18 years	Meta-analysis	Community and student samples
Iceland	10.4%	Wijma et al., 2003 [21]	18 years	Cross sectional study	3641 women at gynecology centers
Iceland	17.6%	Gault Sherman et al., 2009 [22]	17 years	Cross sectional national survey	8618 Icelandic youth
Iceland	35.7%	Asgeirsdottir, 2011 [23]	18 years	Cross sectional survey	9085 Icelandic college students
Norway	10.2%	Thoresen et al., 2015 [24]	18 years	Cross sectional survey	2435 women 2092 men aged 18–75
Nordic Countries	11–36%	Kloppen et al., 2016 [25]	18 years	Literature review	
Swiss	40.2%	Mohler-Kuo et al., 2014 [26]	16 years	Cross sectional study	6787 ninth grade adolescents
USA	27%	Finkelhor et al., 1990 [27]	18 years	National survey	Adults
USA	32.3%	Briere and Elliott, 2003 [28]	18 years	Random sample	1442 adults
USA	26.6%	Finkelhor et al., 2014 [29]	17 years	National telephone surveys	Adults

**Table 3:** The 12 research steps of the Vancouver-School and steps taken in this study.

Steps in the Research Process	Steps Taken in This Study
Step 1. Selecting dialogue partners (the sample).	One woman was selected through purposeful sampling.
Step 2. Silence (before entering a dialogue).	Preconceived ideas were deliberately put aside.
Step 3. Participating in a dialogue (data collection).	Seven interviews with the participant, the first author conducted all the interviews.
Step 4. Sharpened awareness of words (data analysis).	Data collecting and data analysis ran concurrently.
Step 5. Beginning consideration of essences (coding).	Trying repeatedly to answer the question: what is the essence of what the woman is saying?
Step 6. Deconstruction of the text and constructing the essential structure of the phenomenon from this case (individual case construction).	The main factors of each interview were highlighted, and the most important factors were used as building blocks for an individual case construction.
Step 7. Verifying each case construction with the relevant participant (verification).	This was carried out with the participant after each interview.
Step 8. Constructing the essential structure of the phenomenon from all the interviews (meta-synthesis of the interviews).	Two researchers participated in the data analysis process and made sure the case construction was based on the actual data.
Step 9. Comparing the essential structure of the phenomenon with the data (verification).	To ensure this, all the transcripts were read over again.
Step 10. Identifying the overriding theme that describes the phenomenon (construction of the main theme).	Screaming body and silent healthcare providers.
Step 11. Verifying the essential structure with the participant (verification).	The results and the conclusions were presented to and verified by the participant.
Step 12. Writing up the findings (reconstruction).	The participant is quoted directly to increase the trustworthiness of the findings and conclusions.

**Table 4:** An overview of Anne’s main psychological traumas and main physical health problems.

Anne’s Age	Psychological Traumas	Main Physical Problems
2/3 until 9	Anne’s father raped her when he had the chance	
4	Parent’s divorce	Physical symptoms started. Got very sick when she was sent to her father. Got mumps. Had chronic dizziness.
6		Often sick, as if she had the flu.
9–10	Psychological abuse by her stepfather	Always tired, could always sleep, felt it was very difficult to breathe, to get the deep breath.
10	Raped by her uncle	
12	Raped by her stepfather	Lost her sight and hearing. Ear infections, eardrums perforated. Widespread pain and anxiety.
13–14	Raped by her friend’s father	Suspected appendicitis turned out to be gastritis. Depression, anxiety, colon spasm.
14–15	Went to boarding school. Bullying started at school	Pain, muscle aches, stomachaches, colon spasm. Started to numb her feelings with food, gained 10 kg during one summer, 20 kg in six months.
16–17		Colon spasm. Myositis in all her muscles. Appendectomy. Ovarian cysts and adhesions. Repeated urinary tract infections. Diagnosed with chlamydia. Always pain in right ovary. Acute pain after sex. Flashbacks and violent nightmares.
21	Raped by a relative. Ex-stepfather tried to rape her	Heavy postpartum depression. Suicidal thoughts. Severe abdominal pain.
24–27		Ectopic pregnancies. Pilonidal cyst. Chronic urinary infection. Operated on twice to move adhesions.
30		Operation to move adhesions. Cervical dysplasia. Went through cervical conization. Quit working because of chronic pain.
32–35		Ectopic pregnancies. Insomnia. Fibromyalgia. Serious problems with the ovaries due to ruptured cysts. Arrhythmia.
36		Hysterectomy (uterus removed) because of nodules. Heavy bleeding (menorrhagia), pain and endometrial hyperplasia. Operation due to an ovarian cyst and laparoscopic surgery because of another ovarian cyst on her right ovary. Diagnosed with cancer in ovaries.
39		Para-thyroid adenoma.
